# Natural Killer Cells: Development, Maturation, and Clinical Utilization

**DOI:** 10.3389/fimmu.2018.01869

**Published:** 2018-08-13

**Authors:** Alex M. Abel, Chao Yang, Monica S. Thakar, Subramaniam Malarkannan

**Affiliations:** ^1^Laboratory of Molecular Immunology and Immunotherapy, Blood Research Institute, Blood Center of Wisconsin, Milwaukee, WI, United States; ^2^Department of Microbiology and Immunology, Medical College of Wisconsin, Milwaukee, WI, United States; ^3^Department of Pediatrics, Medical College of Wisconsin, Milwaukee, WI, United States; ^4^Department of Medicine, Medical College of Wisconsin, Milwaukee, WI, United States; ^5^Center of Excellence in Prostate Cancer, Medical College of Wisconsin, Milwaukee, WI, United States

**Keywords:** developmental stages, human, mouse, natural killer cells, effector functions

## Abstract

Natural killer (NK) cells are the predominant innate lymphocyte subsets that mediate anti-tumor and anti-viral responses, and therefore possess promising clinical utilization. NK cells do not express polymorphic clonotypic receptors and utilize inhibitory receptors (killer immunoglobulin-like receptor and Ly49) to develop, mature, and recognize “*self*” from “*non-self*.” The essential roles of common gamma cytokines such as interleukin (IL)-2, IL-7, and IL-15 in the commitment and development of NK cells are well established. However, the critical functions of pro-inflammatory cytokines IL-12, IL-18, IL-27, and IL-35 in the transcriptional-priming of NK cells are only starting to emerge. Recent studies have highlighted multiple shared characteristics between NK cells the adaptive immune lymphocytes. NK cells utilize unique signaling pathways that offer exclusive ways to genetically manipulate to improve their effector functions. Here, we summarize the recent advances made in the understanding of how NK cells develop, mature, and their potential translational use in the clinic.

## Introduction

Experiments aimed at characterizing T cell-mediated cytotoxicity inadvertently uncovered the existence of a naturally occurring cytotoxic lymphocyte with intrinsic and innate anti-tumor properties (1). These original observations were made in the 1960s (2, 3) and, within 10 years, researchers began to explore a previously uncharacterized innate lymphocyte population known today as natural killer (NK) cells (4–7). As their name suggests, NK cells are “naturally” cytotoxic and, in contrast to cytotoxic T cells, do not require prior antigen exposure to mediate their anti-tumor effects (4, 7). NK cell activity was first observed in human peripheral blood mononuclear cells (8, 9) and rodent splenocytes (5, 6); however, these large granular lymphocytes are known to reside in multiple lymphoid and non-lymphoid tissues including the bone marrow (BM), lymph nodes (LNs), skin, gut, tonsils, liver, and lungs (10). In this review, we summarize the established and emerging themes of NK cells related to their development, maturation, effector functions such as cytokine production and anti-tumor cytotoxicity, role in the clearance of viral and bacterial infections, and the clinical utilization of donor-derived or genetically modified NK cells.

## Development and Functional Maturation of NK Cells

Natural killer cells were initially thought to develop exclusively in the BM. However, recent evidence in humans and mice suggests that they can also develop and mature in secondary lymphoid tissues (SLTs) including tonsils, spleen, and LNs (11). The cellular progenitors and intermediate populations that give rise to NK cells are defined by the differential expression of lineage-specific surface markers (12). Although these markers are often different between humans and mice, the developmentally regulated expression of critical transcription factors, such as the T-box transcription factors T-bet and Eomesodermin, control NK cell-specific qualities in both species (13).

Natural killer cells represent 5–20% of circulating lymphocytes in humans (14). The percentages of NK cells among lymphocytes ranges between about 2–5% in the spleens and BMs of inbred laboratory mice (15) and about twice that number in wild-caught mice (16). They are distinguished by their unique functions and expression of surface antigens. NK cells lack the clonotypic T cell receptor (TCR) of T and NKT cells and its associated signal-transducing adaptor, CD3ε. In humans, subsets of NK cells express the activating Fc receptor, CD16 and most express CD56 [neural cell adhesion molecule (NCAM) or Leu-19] (17, 18). In C57BL/6 mice, NK cells are identified by the presence of NK1.1 (NKR-P1C) and NCR1 (NKp46/CD335), as well as CD49b (DX5, Integrin VLA-2α), are common NK cell markers in other mouse backgrounds (19, 20). NK cells are most similar to a group of lymphocytes known as innate lymphoid cells (ILCs) (21). ILCs are further categorized into three distinct groups and are present in both humans and mice (11, 21). NK cells are related to group 1 ILCs as both produce interferon-gamma (IFN-γ) and tumor necrosis factor (TNF)-α upon stimulation (22). However, unlike Group 1 ILCs, NK cells have cytolytic functions that resemble those of CD8^+^ cytotoxic T lymphocytes (22).

### Developmental Stages of Murine and Human NK Cells

**Figure 1:**
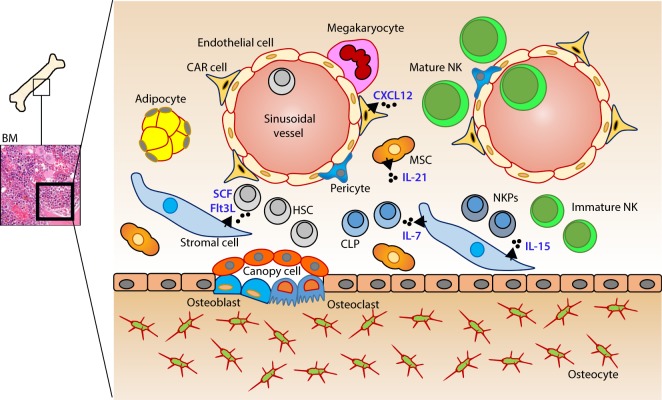
Murine bone marrow niche where natural killer (NK) cells develop. Quiescent hematopoietic stem cells (HSCs) from a hypoxic microenvironment, within the perivascular region proximal to sinusoidal vessels, are induced by hormonal and cytokine cues. Upon unique stimulations [such as stem cell factor (SCF); Fms-like tyrosine kinase-3 ligand (Flt3L)], the self-renewing multipotent HSCs commit to becoming common lymphoid progenitors (CLPs). Non-hematopoietic stromal cells [mesenchymal stromal cells (MSCs), fibroblastic reticular cells] that produce interleukin (IL)-7 or IL-15 play pleiotropic roles in programming CLPs into distinct lymphoid lineages including NK cell progenitors (NKPs). MSCs also produce another common gamma chain receptor (γ_c_R)-binding cytokine, IL-21 that may help with the expansion of the NKPs. CXCL12-abundant reticular (CAR) cells generate CXCL12, which stimulates NKPs *via* CXCR4 to functionally mature the NKPs or immature NK cells (iNKs) into established Mature NK (mNK) cells subsets. mNK cells traffic to secondary lymphoid organs *via* the sinusoidal blood vessels. Other cell types, pericytes, megakaryocytes, adipocytes, canopy cells, osteoblasts, osteoclasts, and osteocytes help form the niche and other supporting systems.

**Figure 2:**
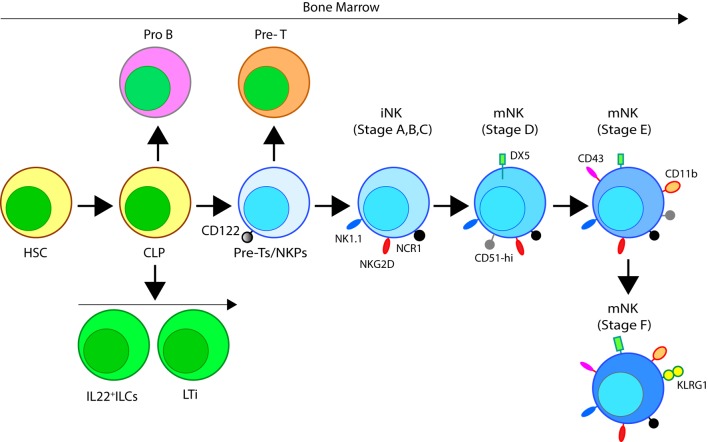
Developmental origin of murine natural killer (NK) cells in the bone marrow (BM). Murine NK cells develop in the BM. A subset of multipotent HSCs commits to becoming oligopotent common lymphoid progenitors (CLPs). CLPs give rise to Pro-B, Pre-T, innate lymphoid cells (ILCs), lymphoid tissue inducers, and CD122^+^ Pre-T/early NK cell progenitor (NKP) lineages. Expression of NKG2D by the CD122^+^ NKPs mark the earliest transition of NKPs into committed immature NK cells (iNK, Stage A). This is followed by the expression of NK1.1 and NCR1 (Stages B and C). Expression of CD51 (Integrin αV) and CD49b (DX5, Integrin VLA-2α) defines the initial stage of mature NK (mNK) cells. Expression of CD43 (Leukosialin), CD11b (Mac-1), and the acquisition of distinct sets of Ly49s define the terminal stage of mNK cells (Stage E). mNK cells migrate into secondary lymphoid organs following the expression of Killer cell Lectin-like Receptor G1 (KLRG1) (Stage F) at least in part by a subset. Additional functional classifications of mNK cells are made using CD27 and CD11b.

In mice, the NK cells develop in specialized BM niches (Figure 1). The hematopoietic niche is most often localized in the perivascular regions proximal to sinusoidal vessels. The multipotent self-renewing hematopoietic stem cells (HSCs) are regulated by an integrated cytokine milieu as part of the endocrine, autocrine, and paracrine signaling. HSCs contain transient self-renewing and long-term quiescent populations. HSCs give rise to all leukocytes and red blood cells. A branch of which constitutes the common lymphoid progenitor (CLP). CLPs give rise to Pro-B, Pre-T, innate lymphoid cells (ILCs), lymphoid tissue inducers, and CD122^+^ Pre-T/early NKP lineages. The cellular origin of NK cells in humans and mice can be traced back to oligopotent CLP (23). Expression of interleukin (IL)-7 receptor-alpha (IL-7Rα, CD127) in Lin^−^CD244^+^ cells mark the earliest step in the transition of CLPs into the lymphoid lineage. A subset of this early progenitor defined as pre-NK cell precursors (Pre-NKPs) expresses the IL-2 receptor β chain (CD122) to become NKPs (24) (Figure 2).

**Figure 3:**
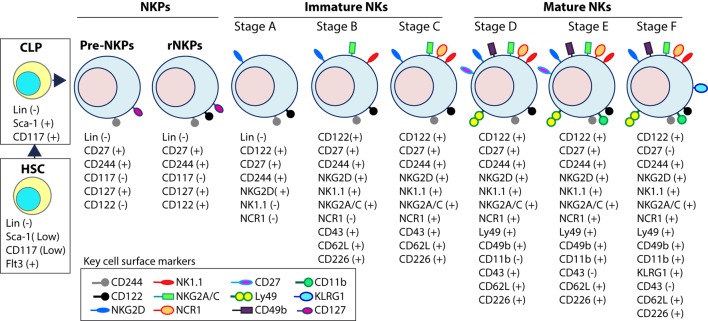
Distinct developmental stages of murine NK cell progenitors (NKPs), immature NK cells (iNKs), and mature NKs (mNKs). Lineage negative (Lin^−^) Sca^+^CD117^+^ hematopoietic stem cells (HSCs) differentiate into common lymphoid progenitors (CLPs) (Lin^−^Sca^Low^CD117^Low^Flt3^+^). Expression of IL-7 receptor-alpha (IL-7Rα) (CD127), CD27, and CD244 mark the full commitment of CLPs into pre-NK cell precursors (Pre-NKPs). Committed NKPs transition from Pre-NKPs to refined-NKPs (rNKPs) by expressing IL-2Rβ (CD122). Expression of NKG2D marks the conversion of rNKPs into iNK cells. Natural killer (NK) cells progressing through the iNK stages express NK1.1 and NKG2A/C followed by NCR1 (Stage A through C). Terminal maturation of iNK cells into mNK cells is defined by the acquisition of distinct sets of Ly49s that help to identify distinct subsets (Stage D). NK cells that have reached terminal maturation downregulate CD27 and express CD11b (Stage E) followed by Killer cell Lectin-like Receptor G1 (KLRG1) (Stage F) by a subset of matured NK cells.

Expression of the activation receptor complex NKG2D/DNAX-activating protein of 10 kDa (DAP10) defines Stage A (Figure 3) of immature NK (iNK) population (25, 26). NKP maintenance and progression to the iNK cell stage requires the activation of transcription factors including an inhibitor of DNA binding 2 (Id2) (27–29) and E4-binding protein 4 (30, 31). By the iNK stage, NK cells express receptors including, NKG2A, DNAM-1 (CD226), NK1.1 (Stage B), and NCR1 (Stage C) as well as the cell adhesion molecules, L-selectin (CD62L) and Leukosialin (CD43) (32). Expression of CD51 (Integrin αV) and CD49b (DX5, Integrin VLA-2α) defines the initial stage (Stage D) of mature NK (mNK) cells. Terminally mNK cells are identified based on the expression of CD43 (Leukosialin) and CD11b (Mac-1). The acquisition of distinct sets of Ly49 receptors also define mNK cells (Stage E) that are functionally licensed (33). In C57BL/6 mice, these inhibitory or activating Ly49s include Ly49A, Ly49C/I, Ly49G or Ly49D, and Ly49H, respectively. mNK cells migrate into secondary lymphoid organs following the expression of Killer cell Lectin-like Receptor G1 (KLRG1) (Stage F) at least in part by a subset (10, 34). NK cells that have reached terminal maturation are fully functional; however, evidence suggests that their capabilities with regards to anti-tumor cytotoxicity and inflammatory cytokine production may not be acquired equally (35, 36).

Functional NK cell maturation can be defined by the differential surface expression of CD27 and CD11b (Mac-1) whereby NK cells develop consecutively through a three-stage program (37). NK cells begin expressing neither receptor, known as the double-negative population, and progress to CD27^+^CD11b^−^ (Stages B, C, and D), double-positive (DP, Stages E), and the CD27^−^CD11b^+^ (Stage F) NK cells, which are considered the most mature (33, 37). Lack of signaling molecule PLC-g2 but not PLC-g1 significantly reduced the terminal maturation of NK cells (38). mNK cells express the activation receptor, CD49b (33), and acquire KLRG1, an inhibitory receptor and marker of terminal maturation (39, 40). Interestingly, DP NK cells have increased effector responses compared to CD27^−^CD11b^+^ NK cells, which suggests the acquisition of regulatory mechanisms during the NK cell maturation process (36).

**Figure 4:**
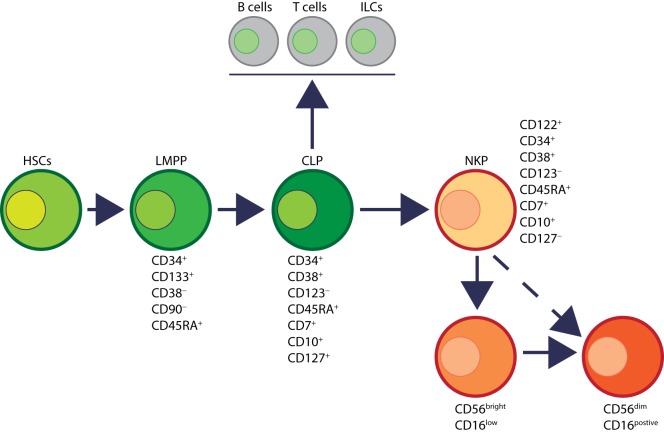
Developmental origin of human natural killer (NK) cells. In human, the primary organ where NK cells mature is still under active investigation. There is ample evidence that NK cells can mature from the lymph nodes (LNs). Lin^−^CD34^+^ hematopoietic stem cells (HSCs) differentiate into CD45RA^+^ lymphoid-primed multipotential progenitor (LMPP). By expressing CD38, CD7, CD10, and the cytokine receptor CD127 (IL-7 receptor-alpha), LMPPs transition into common lymphoid progenitors (CLPs) that have the potential to make lineage commitment into Pro-B, Pre-T, NK cell progenitors (NKPs), or other innate innate lymphoid cells. Expression of CD122 (IL-2Rβ) marks the irreversible fate decision of CLPs into NK lineage. The appearance of CD56 (neural cell adhesion molecule) indicates a final transition of immature NK cell (iNK) into mature NK cells. Most of the iNK cells transition into a minor CD56^bright^ population (~5%) that convert into major CD56^dim^ (>90%) population. It is also suggested that iNK cells can directly give rise to CD56^dim^ population (dotted arrow) that is yet to be validated.

Human NK cells have been shown to mature in the BM and secondary lymphoid organs such as LNs (11, 41). Lin^−^CD34^+^CD133^+^CD244^+^ HSCs differentiate into CD45RA^+^ lymphoid-primed multipotential progenitor in Stage 1 (LMPP, Figure 4). CD34 is a highly glycosylated cell membrane protein and a marker for stemness that facilitates the adhesion of stem cells to the extracellular matrix (42). CD133 is a glycoprotein known as Prominin-1 (43, 44) and CD244 (2B4) is a SLAM family member (45). By expressing CD38 (cyclic ADP ribose hydrolase) (46), CD7 (Ig family, co-stimulatory molecule) (47), CD10 (neutral endopeptidase) (48), and the cytokine receptor CD127 (IL-7Rα), LMPPs transition into CLPs with potential to make lineage commitments into Pro-B, Pre-T, NKPs, or other innate lymphoid cells (ILCs) (49). Expression of CD122 (IL-2Rβ) marks the irreversible fate decision of CLPs into NK lineage. The appearance of CD56 (NCAM) indicates a final transition of iNK into mNK cells. It is also suggested that iNK cells can directly give rise to CD56^dim^ population (dotted arrow) that is yet to be validated (50) (Figure 4).

**Figure 5:**
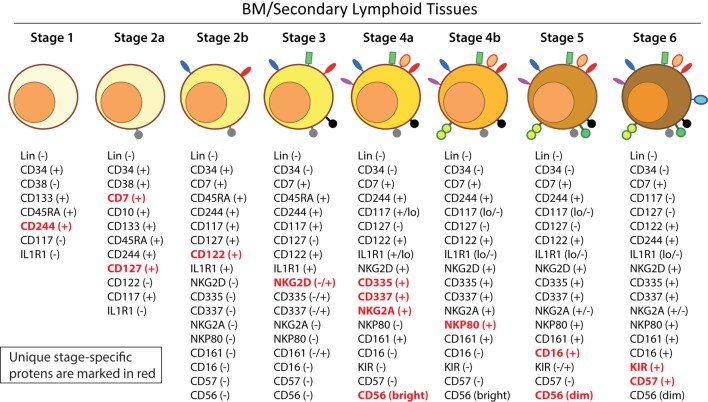
A common schema of human natural killer (NK) cell development in the bone marrow and lymph nodes. A total of six distinct developmental stages have been described with Stages 2 and 4 having additional bifurcations. Similar to the mouse, human NK cells express CD244 (2B4) throughout the developmental process starting at Stage 1 (pre-NK cell precursors). CD117 (c-Kit) and the low levels of interleukin (IL)-1R1 expressions define the Stage 2a and Stage 2b, respectively (NK cell progenitors). A higher expression of IL-1R1 defines the Stage 3 [immature NK cell (iNK)], and the expressions of NKG2D, CD335 (NKp46), CD337 (NKp30), and CD161 (NK1.1) are initiated. Stages 4a and 4b defines an entry of iNKs into mature nks, and are differentiated by the expression of NKp80 at the Stage 4b. Expressions of NKG2D, CD335, CD337, and CD161 reach their maximal levels at Stage 4. Most important of all, CD56 expression peaks (CD56^bright^). Significant differences between Stage 4b and Stage 5 are defined by a decrease in the expression of CD56 (CD56^dim^) in most and initiation of the expression of CD16 (FcγRIIIA) and killer immunoglobulin-like receptor (KIR) (CD158) in a subset of NK cells. Stage 6 defines the generation of “adaptive” or “memory-like” NK cells following “antigen” exposure, and it is identified by the high levels of NKG2C.

Distinct stages through which human NK cells develop are less understood compared to that of the murine counterparts (51). Recent work has helped to demarcate a total of six stages of human NK cell development (Figure 5) based on their both BM and LN development (11, 41). CD3ε^−^CD7^+^CD127^+^ cells mark the earliest stage of committed NKPs (Stage 2a). CD7, whose expression persists throughout development and in mNK cells is a cell membrane protein that recruits PI(3)K *via* a YEDM motif in its cytoplasmic tail (52). Although discrete subsets of CD7-expressing (low and high) CD8^+^ T cells (53) have been described, similar distinctions are yet to be identified in NK cells. Expression of IL-1R, a receptor for IL-1β defines Stage 2b. Expression of activation receptors including NKG2D (CD314, C-type lectin-like, KLRK1), CD335 (Natural cytotoxicity receptor, NCR1, NKp46), and CD337 (NCR3, NKp30) marks the transition of NK cells from Stage 2b to Stage 3. Human NKG2D uses only DAP10 adaptor protein, compared to mouse NKG2D that uses both DAP10 and DNAX-activating protein of 12 kDa (DAP12). NCR1 uses CD3ζ and FcεRγ while NCR3 utilizes CD3ζ as their adaptor complexes. Stage 4 of human NK cell development is sub-divided into two parts based on the expression of the activating receptor NKP80 (KLRF1, type II transmembrane protein) (54, 55). The primary distinction of NK cells in the Stage 4a is that they express abundant amounts of CD56 (CD56^bright^). These NK cells are NKP80^−^ and express the maximal levels of NKG2D, CD335, CD337, inhibitory NKG2A [CD159a, contains two immunoreceptor-based tyrosine inhibitory motifs (ITIMs)] and CD161 (NK1.1, KLRB1, NKR-P1A). At Stage 4b, human NK cells become positive for NKP80 and maintain their CD56^bright^ status.

Downregulation of CD56^bright^ expression to become CD56^dim^ in most and the expression of immunoglobulin superfamily member CD16 (FcγRIII) in a subset of NK cells defines Stage 5 (Figure 5). Similar to the CD27/CD11b classification in mouse, expression levels of CD56 provides a functional classification of human NK cells. Most human NK cells in the peripheral blood are CD56^dim^ (56). CD56^bright^ NK cells are considered less mature and reside primarily in SLTs while the CD56^dim^ subset represents the majority of NK cells in circulation (57). Most of the iNK cells transition into a minor CD56^bright^ population (~5%) that convert into major CD56^dim^ (>90%) population. The downregulation of CD56 during human NK cell maturation is strongly associated with the acquisition of anti-tumor cytotoxicity as CD56^bright^ NK cells are potent producers of inflammatory cytokines, while the cytolytic function of human NK cells resides primarily in the CD56^dim^ population (58, 59). Terminal maturation (Stage 6) of CD56^dim^ NK cells are defined by the expression of CD57 (HNK-1, Leu-7). Additional classification such as “antigen-experienced” or “adaptive” CD2^+^ NK cells is defined by a higher expression of NKG2C (KLRC2, CD159c) (60–63).

### Role of Common Gamma Chain Cytokines in the Development of NK Cells

**Figure 6:**
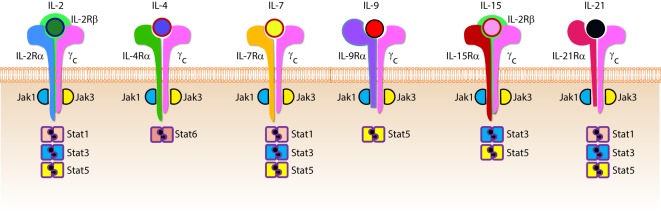
Role of common gamma-containing receptors in natural killer (NK) cell development. The common gamma chain-containing receptor family consists of six members, interleukin (IL)-2R, IL-4R, IL-7R, IL-9R, IL-15R, and IL-21R. Each one of them is distinguished from others by their unique α-chains. IL-2Rβ is shared by IL-2R and IL-15R complexes. In mouse, NK cell progenitors (NKPs) utilize IL-7R early during their transition from Pre-NKPs into refined-NKPs. In human, apart from its role in the early development, IL-7 also regulates the survival and expansion of mature CD56^bright^ NK cells. IL-15R and IL-21R are required by NK cells to initiate and sustain their proliferation. Although it has been widely used to expand human and mouse NK cells *ex vivo*, the *in vivo* role of IL-2 that is primarily produced by CD4^+^ T cells is yet to be better understood. Role of IL-4 and IL-9 in NK cell development is less explored. Distinct sets of Janus kinases (JAK) and signal transducers and activators of transcription (STAT) associate and transmit the signaling from the common gamma chain-associated cytokine receptors.

Cytokines are essential inflammatory mediators that control multiple aspects of NK cell biology. NK cells express cytokine receptors early in their development (26) and require signaling through the common gamma (γ_c_) chain for their development, homeostasis, and function (64). The γ_c_ chain (CD132) is a 40 kDa type I transmembrane glycoprotein that serves as the signaling subunit for IL-2, IL-4, IL-7, IL-9, IL-15, and IL-21 (65). Although these cytokines display some functional redundancy, their cell-specific functions during an immune response are determined by the expression of distinct receptor complexes (Figure 6). For instance, IL-4, IL-7, IL-9, and IL-21, bind to high-affinity receptor complexes consisting of a cytokine-specific alpha-chain and the γ_c_ (64). These receptors have no intrinsic kinase activity, so signal transduction in response to γ_c_ cytokines is initiated by receptor-associated Janus kinases (JAKs) which phosphorylate different STAT molecules in a cytokine-dependent manner (66, 67).

Interleukin-2 and IL-15 are functionally related members the γ_c_ family of cytokines with respect to their receptor interactions as both can signal through complexes consisting of the γ_c_ and IL-2Rβ chains (68) resulting in the activation of STAT1 and STAT5 *via* JAK-1 and JAK-3, respectively (69). However, cellular affinity for either IL-2 or IL-15 is altered by the expression of high-affinity heterotrimeric complexes containing IL-2 or IL-15-specific alpha subunits (64). IL-2Rα (CD25) is expressed on activated NK cells and substantially increases their affinity for IL-2 which drives their proliferation and production of lytic molecules such as perforin and Granzyme B (70). Given that NK cells are found near T cell areas in SLTs (10), T cell-derived IL-2 may facilitate a vital functional crosstalk between innate and adaptive lymphocytes during an infection (71).

Although NK cells require γ_c_ signaling, as evidenced by the significant reduction in NK cell number and functional impairment in mice lacking the γ_c_ chain (γ_c_^−/−^) (72, 73), IL-15 is unique in this regard. Mice lacking IL-15, IL-15Rα, or IL-2Rβ have similar phenotypes to γ_c_^−/−^ mice with respect to NK cell deficiencies (74–76), and transgenic overexpression of IL-15 in mice results in increased NK cell generation (77). It was determined that IL-15-mediated proliferation of mouse T cells was dependent on the presence of IL-15Rα on surrounding cells (78) which revealed a trans-presentation mechanism that is not required for IL-2-mediated proliferation. For this to occur, soluble IL-15 binds to IL-15Rα on the surface presenting cells which trans-present this complex to apposing NK cells expressing IL2-Rβ/γ_c_ heterodimers (79). IL-15 can be trans-presented by dendritic cells (DCs) and macrophages as well as non-hematopoietic cells including stromal cells and epithelial cells (80). The importance of IL-15 trans-presentation for NK cell survival *in vivo* was demonstrated with adoptive transfer experiments that showed normal NK cells were unable to survive in IL-15Rα-deficient mice while NK cells lacking IL-15Rα persist in IL-15Rα-sufficient recipients (81). IL-21R utilizes IL-21Rα and the γ_c_ (82). IL-21 synergizes with IL-2 to augment the expression of NKG2A, CD25, CD86, CD69, Perforin, and Granzyme B and thereby augmented cytotoxicity (83). These cytokines that use the γ_c_-based receptors are the obligatory link between NK cells and the cells that produce them. For example, T helper cells that produce IL-21 can regulate the expression levels of activation receptors or cytolytic contents in NK cells. Similarly, DCs that produce IL-15 plays an essential role in the proliferation and priming of NK cells (discussed in detail elsewhere in this review).

### Educating NK Cells to Distinguish “*Self*” From “*Non-Self*”

Functional differences between NK cells is also a consequence of the NK cell education process through which NK cells interact with self-major histocompatibility complex (MHC)-I (84). Initial observations concerning hybrid resistance to NK cell-mediated transplant rejection demonstrated that F_1_ hybrid mice reject transplanted BM from either parent while they do not reject transplants from other F_1_ mice (85, 86). These studies, along with others utilizing β2-microglobulin-deficient mice, further revealed that the underlying mechanistic basis of this rejection was dependent on MHC-I surface expression (87). The NK cell receptors that interact with MHC-I belong primarily to the killer immunoglobulin-like receptor (KIR) family in humans and the lectin-like homodimeric Ly49 receptor family in mice, and it is through these receptors that MHC-I regulates NK cell function (84). The molecular basis of NK cell education is still under debate and, based on the “*missing-self*” hypothesis of NK cell activation, it was initially thought that self-tolerance was exclusively due to inhibitory receptor signaling upon MHC-I engagement when interacting with normal cells (88). However, there exists a relatively small population of NK cells that do not express self-reactive inhibitory receptors under normal conditions, and these cells are hypofunctional upon stimulation (89).

The use of transgenic mouse models has led to the prevailing theories that attempt to explain the NK cell education process. In 2005, Yokoyama and colleagues termed the widely accepted model of NK cell education as “*licensing*” (90) which proposes that phosphatase activation in response to the ITIMs found in inhibitory receptors ultimately controls NK cell responsiveness. Thus, licensed NK cells are deemed functionally competent and are self-tolerant due to the interaction between inhibitory receptors and MHC-I while unlicensed NK cells, represented by those that do not express self-MHC-I-specific inhibitory receptors, are tolerant because they are functionally incompetent (84).

To further explain how NK cells become educated or “*licensed*,” Raulet and Vance proposed the NK cell “*arming*” and “*disarming*” models (91). In the “*arming*” model of NK cell education, NK cells are deemed functionally mature through self-MHC-I-specific inhibitory receptor interactions which are sufficient to drive the NK cell education process. This may seem counterintuitive given that these receptors are known to be exclusively inhibitory; however, their designation as such was described with respect to NK cell effector functions (91). Thus, inhibitory receptors may possess alternative functions in terms of NK cell education, and it has been demonstrated that signaling through these receptors is likely more complicated than previously appreciated (92). The “*disarming*” model proposes that chronic stimulation of NK cells that lack self-MHC-I inhibitory receptors are rendered hyporesponsive to stimulatory receptor activation potentially through a process similar to anergy in T or B cells (91). While these processes are thought to control NK cell responsiveness primarily during development, new interpretations of these models suggest that they may be altered under disease conditions and function as a rheostat to set the threshold of NK cell activation in the periphery (93, 94). Overall, the molecular mechanisms that regulate NK cell education have yet to be described though it is clear that the NK cell education process dictates their functional capabilities.

### Signaling in NK Cells: Role of Germline-Encoded Activation Receptors

Natural killer cells do not express clonotypic receptors. However, they mediate strong anti-tumor cytotoxicity and generate significant quantities of pro-inflammatory cytokines (95). Lack of variable clonotypic receptors is compensated by multiple germline-encoded NK cell activation receptors (NKRs) such as NKG2D, NCR1, NCR2, NCR3, NKG2C, CD244, Ly49D, and Ly49H. Expression of more than one NKR that recognize self or pathogen-derived ligands endows NK cells with inherent, innate abilities to mediate effector functions. Due to the expression of multiple activation receptors, NK cells have to follow a distinct developmental program to obviate misrecognition of “*self*” leading to autoimmune responses. The varied nature of NKRs and the absence of signaling domains in their cytoplasmic tails necessitates the association and recruitment of receptor-associated adaptor molecules for signal transduction (96). The adaptor molecules that propagate NKR signaling includes FcεRIγ, CD3ζ, and the DAP12 which signal *via* immunoreceptor tyrosine-based activation motifs (ITAMs) contained within their cytoplasmic domains. NKRs that utilize these signaling adaptors include CD16, NCR1, Ly49D, Ly49H, and NKG2D (97–101). However, Ly49H and NKG2D can also signal *via* the YINM motif present within the adaptor, DAP10 (101–103). NK cell activation through these receptors occurs by interacting with distinct cellular and foreign ligands present on diseased cells and form the basis for the NK cell-mediated immune response in multiple contexts.

NKG2D is a homodimer forming C-type lectin-like type II transmembrane glycoprotein that is highly conserved from mice to humans (104). NKG2D is constitutively expressed on NK cells (105) and recognizes stress-inducible ligands that are structurally related to MHC-I (104). These ligands include ULBPs (106–108), MIC-A (109), and MIC-B (110, 111) in humans, and H60 (112–115) (a, b, and c), Rae-1 (α-ε) (115–117), and Mult1 (118, 119) in mice (120). NKG2D signaling is mediated through DAP10 and DAP12 *via* YINM and ITAM tyrosine-based signaling motifs, respectively. DAP10 recruits and activates the p85α subunit of PI(3)K (121) and recruits Grb2 (105) while DAP12 recruits ZAP70 and Syk to initiate NKG2D-mediated NK cell activation (105, 122).

These receptor-proximal signaling molecules activate the CBM signalosome containing Carma1, Bcl10, and Malt1, as well as Akt and the MAPKs, Erk1/2, Jnk1/2, and p38 (123–125). NK cell activation through NKG2D results in the mobilization of lytic granules as well as cytokine production *via* activation of transcription factors including activator protein-1 (AP-1) and NF-κB (123, 124). Pharmacological or genetic inhibition of these pathways causes deficiencies in NK cell-mediated cytotoxicity and pro-inflammatory cytokine production (126, 127). Pro-inflammatory cytokine production from NK cells expressing a catalytically inactive form of PI(3)K-p110δ^D910A^, was significantly reduced while anti-tumor cytotoxicity was only moderately impaired (128–131). This finding substantiates the notion that the signaling molecules required for NK cell effector functions are not mutually exclusive (124) and further investigation is required to fully elucidate the molecular mechanisms that regulate NK cell effector functions in response to NKG2D-mediated stimulation.

## NK Cell Effector Functions

Natural killer cells mediate their immunomodulatory effects through two critical effector functions. First, NK cells are cytotoxic lymphocytes that can directly lyse cells that have undergone a malignant transformation or have become infected with a virus or other intracellular pathogen (22). The cytolytic function of NK cells can initiate through a variety of processes, including degranulation and death receptor ligation, and is critical for the clearance of diseased and dysfunctional cells (132, 133). Second, NK cells can produce a variety of inflammatory cytokines in response to activation receptor stimulation as well as inflammatory cytokine-induced activation signaling (134, 135). These NK cell effector functions are essential components of the immune response and are the primary mechanisms through which NK cells mediate protective immunity.

### The Mechanisms That Facilitate NK Cell Cytotoxicity

**Figure 7:**
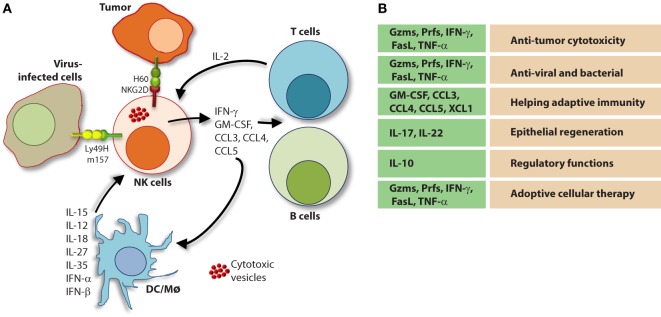
Role of a “third signal” in natural killer (NK) cell activation. **(A)** A brief description of the significant interactions between NK and myeloid cells. NK cells possess inherent abilities to mediate cytotoxicity and produce inflammatory cytokines and chemokines. Myeloid cell-derived cytokines play a central role in regulating the effector functions of NK cells. Interactions between the innate NK cells and the primary arms of the adaptive immunity (T and B cells) are less explored. Stimulation through activation receptors (i.e., NKG2D or Ly49H) help recognize tumor (H60) or infected target cells (murine cytomegalovirus-derived m157). **(B)** A summary of major soluble factors produced by NK cells and their intended functions.

The molecular mechanisms that regulate NK cell cytotoxicity have been well described and can be divided into three main processes: (1) target cell recognition, (2) target cell contact and immunological synapse (IS) formation, and (3) NK cell-induced target cell death. Distinct mechanisms have been described for how target cells are recognized by NK cells and how they deem diseased cells appropriate for destruction (Figure 7). Once recognized, NK cells directly interact with the target cell of interest through the formation of a lytic IS which facilitates NK cell-induced target cell death through two essential mechanisms (136).

The first mechanism involves the activation of death receptors present on the surface of the target cell which initiates the extrinsic apoptotic pathway (137). These receptors include TNF-related apoptosis-inducing ligand-receptor (TRAIL-R) and Fas (CD95) which are activated by their cognate ligands, Fas ligand (FasL) (CD95L) and TRAIL, present on NK cells (133). The surface expression of death receptors can be induced on target cells by NK cell-derived IFN-γ (138), and their activation initiates many pro-apoptotic signaling programs (139, 140). The death receptor superfamily is characterized by the utilization of a cytoplasmic death domain which enables these receptors to activate the apoptotic machinery including initiator caspases-8 and 10 (141, 142). Initiator caspases promote a cascade of IL1β-converting enzyme (ICE) superfamily proteases, including caspase-3 (143), and induce mitochondrial damage and cytochrome C release resulting in the formation of the apoptosome (144). The apoptosome amplifies initiator caspase-mediated substrate cleavage and, along with caspase-3-induced DNA fragmentation *via* caspase-activated DNase activation (145), results in cell death *via* apoptosis (146).

The primary mechanism of NK cell-mediated cytotoxicity involves the directed release of lytic molecules to the target cell (147). NK cells store these molecules in cytolytic granules that are delivered to the target cell through membrane fusion at the IS (136). This process requires cytoskeletal reorganization events including actin polymerization at the IS (148, 149) as well as polarization of the microtubule organizing center toward the target cell (150). Polarized lytic granules travel along microtubules and, once at the IS, fuse with the target cell membrane and release enzymes that facilitate that activation of the intrinsic apoptosis program within the target cell (136, 151). The molecules contained within lytic granules include the 60–70-kDa pore-forming glycoprotein, perforin (152), class of serine proteases known as granzymes (133), FasL (CD178), TRAIL (CD253), and granulysin (153). Granzyme B and perforin are a critical component of NK cell lytic granules and is classified as an apase that cleaves peptides after aspartic acid residues (133). Once inside the target cell, Granzyme B can trigger apoptosis through caspase-dependent and independent mechanisms. Granzyme B activates caspase-dependent apoptosis at multiple points in the apoptotic pathway by directly cleaving the apoptotic initiator caspase-8 as well as caspase-3 (154, 155). Granzyme B can also induce apoptosis in a caspase-independent manner and induce cytochrome C release from the mitochondria through the proteolytic cleavage of the pro-apoptotic protein, Bid (156).

### NK Cell-Mediated Pro-Inflammatory Cytokine Production

Natural killer cells are potent producers of pro-inflammatory and immunosuppressive cytokines. However, the release of inflammatory cytokines is distinct from cytotoxic granule secretion (157) and NK cells utilize activation-induced signaling components to differentially regulate these two functions (124). Although NK cells can produce a wide-range of cytokines depending on the inflammatory environment (158, 159), NK cells primarily produce Th1-type cytokines when responding to tumor ligands and intracellular pathogens (160, 161). These include IFN-γ, TNF, and granulocyte/monocyte colony-stimulating factor (GM-CSF) which facilitate the activation of T cells as well as other innate immune mediators such as DCs, macrophages, and neutrophils (162, 163). NK cells also produce chemotactic cytokines (chemokines) including CCL3 (MIP-1α), CCl4 (MIP-1β), CCL5 (RANTES), XCL1 (lymphotoxin), and CXCL8 (IL-8) which can attract effector lymphocytes and myeloid cells to inflamed tissues (164).

Transcriptional activation of cytolytic molecules and inflammatory cytokines is a highly regulated process mediated by a variety of transcriptional regulators in NK cells. Many of these transcription factors, such as T-bet, are lineage defining and become activated early in NK cell development (13). Cytokine-induced activation of transcription factors, such as signal Transducers and Activators of Transcription (STAT) 4 and 5, occurs in response to IL-12 and IL-2 + IL-15 signaling, respectively (165). NKRs also initiate inflammatory transcriptional programs upon activation. These include the c-Fos and c-Jun heterodimer, AP-1, nuclear factor kappa-light-chain-enhancer of activated B cells (NF-κB), and nuclear factor of activated T cells (124, 166, 167) which bind promotor regions and promote inflammatory cytokine gene transcription (168, 169).

### Role of Pro-Inflammatory Cytokines that Provide a “Third Signal” to NK Cells

A variety of cells generate a number of inflammatory mediators to sensitize and prime NK cells. Among these DCs play a central role (170). A complex interplay between DCs and NK cells is defined as one of the critical steps for the sensitization of NK cells (171). Given DC generate critical cytokines such as IL-15, IL-12, IL-23, IL-27, and IL-18, the crosstalk with NK cells determines the pathophysiological outcome of an ongoing immune response (172). Priming with type-1 IFN-α/IFN-β results in the expression of IL-15Rα and generation of IL-15 from plasmacytoid DCs (171). Multiple cell types including NK cells produce type-1 IFNs by which they can prime DCs (173). The trans-presentation of IL-15 by IL-15Rα to IL-15Rα/IL-2Rβ/IL-2Rγ complex on NK cells initiates multiple cellular tasks including proliferation and transcriptional reprogramming (81, 174). The IL-12 family of heterodimeric cytokines includes IL-12, IL-23, IL-27, and IL-35 which mediate diverse functions in NK cells (Figure 7) (175). IL-27 has both activating and inhibitory functions (176, 177) and IL-35 is an immunosuppressive cytokine produced exclusively by regulatory T cells (178). IL-12 and IL-23 are both produced by pathogen-activated macrophages and DCs and share a common component of their heterodimeric receptors, IL-12Rβ1 (175). Although the function of IL-23 in NK cells remains under debate, the role of IL-12 in NK cell activation is well established (175). IL-12 is a combination of the p40 and p35 alpha and beta subunits, respectively, and binds the IL-12 receptor (IL-12R) complex, IL-12Rβ1/IL-12Rβ2 (179). IL-12R signaling is propagated by Tyrosine kinase 2/JAK-2 and activates the transcriptional regulator, STAT4 (180).

Interleukin-12 signaling synergizes with those of other cytokines, including IL-2, IL-15, and IL-18 significantly enhances IFN-γ production by NK cells (181). IL-18 is a member of the IL-1 cytokine family and signals *via* the IL-18 receptor (IL-18R) through the signaling adaptors, myeloid differentiation primary response 88, and IL-1R-associated kinase (182, 183). IL-18 alone is not sufficient to induce IFN-γ production; however, the expression of IL-18R is induced by IL-12-mediated activation in lymphocytes (184) and IL-18 signaling synergizes with IL-12-mediated stimulation. Specifically, STAT4 activation by IL-12 enhances *Ifng* gene transcription while IL-18R signaling simultaneously induces the promoter binding activity of AP-1 and activates p38 MAPK to promote *Ifng* transcript stability and IFN-γ protein production (185, 186).

## NK Cells in Health and Disease

To date, the diverse functions of NK cells in mammalian immunity is not fully understood. However, accumulating data collected from patients with rare disorders characterized by NK cell deficiency have shed light on their relevance to human health (187) and studies using genetically modified mouse models have generated intriguing ideas with regards to their pro-inflammatory and immunosuppressive functions (188). NK cells produce and respond to inflammatory stimuli and are most well known for their roles in anti-viral immunity and tumor immunosurveillance; however, NK cells are also involved in a variety of autoimmune disorders as drivers of pathologic inflammation (189). Emerging evidence also demonstrates that NK cells can regulate anti-inflammatory programs, such as tissue repair (190, 191). Whether NK cells act as primary innate effectors or accessory cells as part of the adaptive immune response appears to be context-dependent, but their contribution as first-line responders and essential inflammatory mediators is well established. Importantly, how the crosstalk between NK cells and lymphocytes (αβ-TCR^+^ T, γδ-TCR^+^ T, NKT, and B cells), myeloid cells (monocytes, macrophages, and DCs), or non-immune cells (epithelial or endothelial cells) enumerate a productive immune response is far from fully understood.

### NK Cell Functions During Viral and Bacterial Infections

Natural killer cells are critical for defense against a wide variety of pathogens. Pattern recognition receptors (PRRs) recognize pathogen-associated molecular patterns and are essential components of the NK cell-mediated innate immune response (192). Activation of NK cells through PRRs elicit the production of TNF and IFN-γ which contribute to antibacterial defense (192, 193). NK cells also contribute to antifungal immunity by direct and indirect mechanisms (194). First, NK cells can directly damage fungal membranes through the targeted release of cytotoxic granules containing the membrane disrupting protein, perforin (195). They can also facilitate the antifungal host response through direct phagocytosis as well as the production of inflammatory mediators (196). Specifically, the production of GM-CSF by NK cells is critical for controlling *C. albicans* infection by promoting the fungicidal activity of neutrophils (197). However, the direct contribution of NK cells to microbial immunity has best been described with regards to their discrete actions against intracellular pathogens.

**Figure 8:**
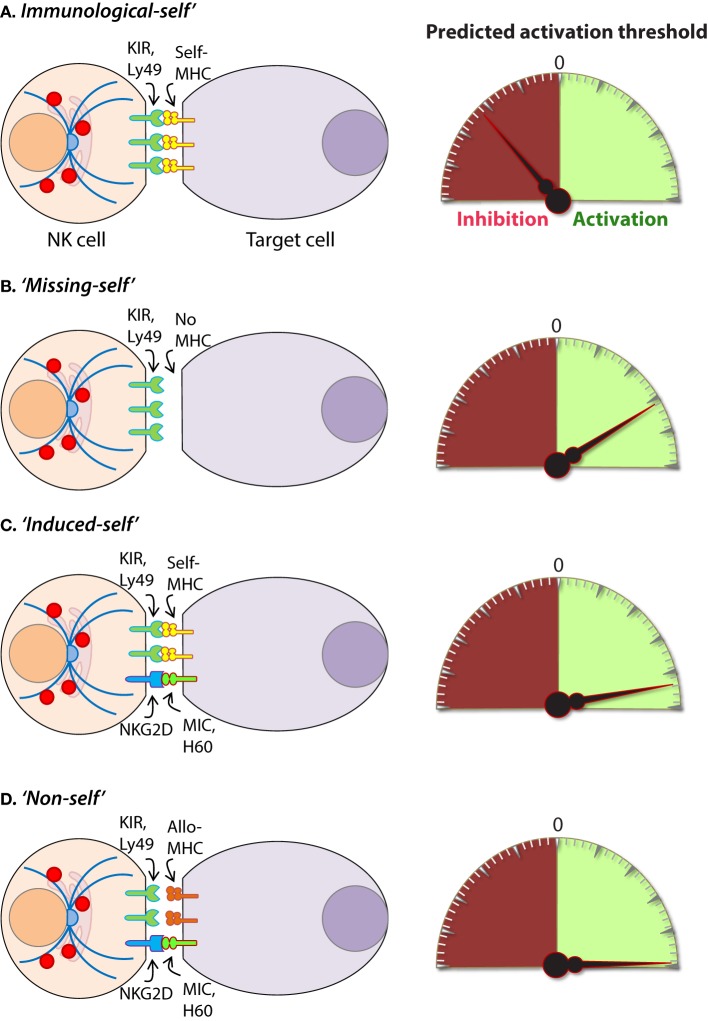
Mechanisms of target cell recognition by natural killer (NK) cells. NK cells lack clonotypic receptors and rely on germline-encoded activation and inhibitory receptors to recognize other cells around them. The following are some of the primary mechanisms by which NK cells perceive target cells. **(A)** “*Immunological Self*”: recognition of autologous MHC class I (MHC-I) (human leukocyte antigen (HLA)) or histocompatibility antigen-2 (H2, mouse) by inhibitory receptors [killer cell immunoglobulin-like receptor (KIR) or Ly49] let the NK cells know that they are interacting with normal cells and contain their activation. **(B)** “*Missing-self*”: recognition of target cells that either does not express MHC-I or reduce them below optimal levels can induce NK cell activation. **(C)** “*Induced-self*”: recognition of activating ligands that are expressed on target cells by the germline-encoded receptors such as NKG2D (H60, mouse; MIC-A/B, human), Ly49H (murine cytomegalovirus-derived m157, mouse), NCR1 (a number of viral proteins) can overcome MHC-I-mediated inhibitory signaling resulting in NK cell activation. **(D)** “*Non-self*”: recognition of transplanted tissue by NK cells, where the donor tissue expresses either allogeneic or haploidentical MHC-I.

Intracellular pathogens have evolved a variety of mechanisms to evade the host immune response including subversion of the MHC immunosurveillance system (198). MHC molecules are highly polymorphic within a population and are encoded by human leukocyte antigen (*HLA*) genes in humans and, *H-2* in mice (199). MHC molecules can be divided into two major classes, MHC class I (MHC-I) and MHC class II (MHC-II). MHC-I molecules bind, and present endogenous peptides to cytotoxic CD8^+^ T cells and subversion of this immunosurveillance mechanism results in an insufficient adaptive immune response (200). MHC-II is abundantly expressed on antigen-presenting cells (APCs) and facilitates the presentation of exogenous peptides to CD4^+^ helper T cells (201). Nearly all somatic cells express endogenous peptides on their surface in the context of MHC-I, and this allows the immune system to sample the intracellular environment (201). The peptide–MHC-I complex also defines the immunological “*self*” condition and maintenance of this system is essential for both immune tolerances as well as the rejection of “*non-self*” cells (Figure 8) and tissues that express distinct MHC-I haplotypes (202).

Natural killer cells possess unique mechanisms to contain intracellular pathogens including viruses and some species of bacteria by lysing infected cells, releasing them and exposing them to adaptive cell-mediated immunity (203, 204). NK cells also produce inflammatory cytokines, such as IFN-γ to contain viral or bacterial growth (205–207). For example, hemagglutinin, a sialic acid receptor expressed by the influenza virus, serves as an activating ligand for NCR1 (208, 209). The murine cytomegalovirus (MCMV)-encoded membrane glycoprotein, m157, is recognized by the Ly49H receptor expressed in C57BL/6-derived NK cells (210). NK cells from other mouse backgrounds, such as 129/SvJ and BALB/c, do not express Ly49H, or another resistance factor, which renders them susceptible to MCMV as they are unable to mount a specific NK cell-mediated immune response to the virus (211–213). NKG2D has also involved in NK cell-mediated anti-viral immunity as evidenced by multiple observations in which human and mouse CMV proteins downregulate cellular stress ligands that activate NK cells through this receptor (214–217).

Natural killer cells have the unique ability to identify infected cells without direct engagement of the MHC-I complex (12, 218). Therefore, intracellular pathogens that evade CD8^+^ T cells by interfering with MHC-I surface expression remain vulnerable to NK cell-mediated immunity (219). In terms of anti-viral immunity, NK cells and CD8^+^ T cells have long been considered to represent the innate and adaptive arms of the immune response, respectively (220). However, the separation of these cells with regards to their contributions to adaptive immunity has recently been reconsidered due to the discovery of NK cells that exhibit immunological memory (160, 221). Although they do not utilize clonotypic receptors, such as the TCR, a relatively small population of memory NK cells has been described as long-lived effectors capable of rapid recall responses (222).

The formation of memory NK cells has been extensively investigated in mice infected with MCMV and studies using this system have been critical in defining the molecules that mediate this phenomenon (222–225). A vaccination study using antigens from viruses including, influenza, vesicular stomatitis virus, and human immunodeficiency virus type 1 also showed memory-like NK cell responses in mice (226) and NK cells exhibited enhanced protection against secondary infections with vaccinia virus and herpes simplex virus type 2 (227, 228). Collectively, these studies provide compelling evidence demonstrating the functional relevance of NK cell memory as a universal anti-viral immune mechanism. Observations in humans have also suggested the ability of human NK cells to form memory (229, 230); however, the full contribution of memory NK cells to anti-viral immunity and potential implications this may have on vaccine development has yet to be determined.

**Figure 9:**
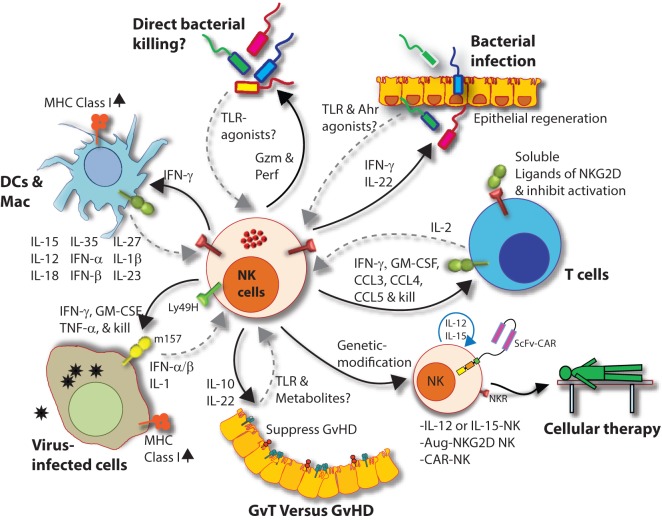
Natural killer (NK) cells in health and disease. As the largest lymphocyte population representing innate immunity, NK cells perform diverse functions. Through their ability to mediate killing and to produce soluble factors, NK cells perform multitudes of immunological functions. Counter-clockwise: bidirectional interactions between NK cell and dendritic cells (DCs)/macrophages result in priming. Activated DCs and macrophages generate interleukin (IL)-15, IL-12, IL-18, IL-35, IFN-α, IFN-β, IL-27, IL-1β, and IL-23. These, in turn, activate NK cells to be primed, proliferate, and to produce inflammatory factors and chemokines such as interferon-gamma (IFN-γ), granulocyte/monocyte colony-stimulating factor (GM-CSF), tumor necrosis factor (TNF)-α, CCL3, CCL4, and CCL5. In addition, IFN-γ from NK cells can increase the MHC class I expression and the transcription of genes encoding immuno-proteasomal subunits in these professional antigen-presenting cells and thereby augmenting T cell priming and activation. Similarly, virus-infected cells produce IFN-α, IFN-β, and IL-1β and present either “stress-induced” self-ligands or viral proteins on the cell surface that activate NK cells. A reduction in graft-versus-host disease (GvHD) is mediated through the production of IL-10 by the CD56^bright^CD16^Neg^ NK cell subset and augmentation of GvT is potentiated *via* direct tumor killing by CD56^dim^CD16^Pos^ NK cell subset. In addition, production of IL-22 by NK subsets may help the regeneration of epithelial cells in the mucosal tissues. Irrespective of these observations, the mechanisms by which NK cells are activated to respond during active GvHD/GvT is not fully understood. Genetic manipulation of NK cells has helped to improve the effector functionality and the longevity of human NK cells *in vivo*. Stable integration of gene encoding IL-15 into the genome of NK cells promotes sustained proliferation *via* an artificial autocrine loop. Similarly, integration of gene encoding IL-12 makes this cytokine abundantly available within the microenvironmental milieu and thereby augment the effector functions of NK cells, specifically, the production of IFN-γ. Augmented expression of NK cell activation receptors (NKRs) including NKG2D and NCR1 by genetic engineering increases the anti-tumor cytotoxicity of NK cells. Other studies have shown the expression of single chain variable fragment that forms the core ectodomain of chimeric antigen receptor (CAR) to augments the tumor-targeted killing of NK cells. These genetically modified NK cells provide exciting newer opportunities for cell-based therapies. The bidirectional interaction between NK and T cells results in the regulation of adaptive immunity. IL-2 produced by CD4^+^ Th1 cells play a vital role in the proliferation and expansion of NK cells. Although *in vitro* experiments consistently have provided support toward this notion, the *in vivo* evidence is far from convincing. However, the inflammatory factors produced by NK cells have a significant impact on both CD8^+^ and CD4^+^ T cells. Expression of “*self*” ligands for NKG2D by T cells results in the recognition and killing of T cells by NK cells during GvHD and anti-viral responses. In addition, a cleaved soluble form of these ligands (MIC-A/B) is present in the serum of cancer patients. This, in turn, plays an important role in containing the effector functions of T cells *via* direct binding to the NKG2D receptor expressed on T cells. NK cells recognize bacteria-infected cells (such as epithelial cells) either using toll-like receptors (TLR) or by activated through soluble factors including aryl hydrocarbon receptor (Ahr). This results in the production of IFN-γ and IL-22 that helps with the reduction in bacterial load and regeneration of epithelial cells, respectively. NK cells can also directly mediate the lysis of bacteria using granzymes and perforin.

Natural killer cells also recognize bacteria and bacterial products either directly or from infected cells and professional APCs (Figure 9) (231). Recent work has shown that NK cells can directly release granzymes proteases to initiate disruption of electron transport, generate superoxide anion, and inactivate bacterial oxidative defenses causing the death of *Listeria monocytogenes, Escherichia coli*, and *Mycobacteria tuberculosis* (232–234). In addition, NK cells using Granzyme B mediated the killing of facultative anaerobic bacteria such as *L. monocytogenes* by cleaving essential proteins that are required for protein translation (aminoacyl tRNA synthetases and ribosomal proteins), folding (protein chaperones), and protein degradation (Clp system) (235). Indirect killing and containment of *L. monocytogenes* (236, 237), *Staphylococcus aureus* (238), *Lactobacillus johnsonii* (239), *Mycobacterium tuberculosis* (240), and *Mycobacterium bovis bacille Calmette-Guérin* (241) by NK cells have been described. Mechanisms by which NK cells mediate indirect clearance of bacteria are complex. Substantial evidence suggests that interleukins including IL-12 and IL-18 from monocytes and DCs play a central role (242–244). Role of other inflammatory cytokines such as IL-27 and its cooperation with IL-18, IL-6, and IL-12 during the clearance of bacterial infections have been identified; however, the precise mechanisms by which NK cells evoke the anti-microbial responses are yet to be elucidated (245, 246).

### Anti-Tumor Functions and the Clinical Utilization of NK Cells

The vital role of NK cells in tumor immunosurveillance was recognized soon after their initial characterization (247, 248). NK cells can detect changes in surface expression of self-MHC-I molecules on autologous cells which distinctively qualifies them to detect cells that have undergone malignant transformation (Figure 8) (218, 248). Genomic mutations that arise during the transformation process are reflected by a variety of phenotypic changes which alter the expression of cell surface molecules, including downregulation of the inhibitory “*self*” MHC-I (200, 249). The activity of NK cells against this “*missing-self*” condition has been well described (250, 251) and serves as a critical mechanism through which NK cells facilitate anti-tumor immunity. Transformed cells also express increased numbers of stress-induced molecules on their surface which can be recognized by specific NK cell receptors, such as NKG2D (120, 252). This concept, known as “*induced self*” (Figure 8) recognition (253, 254), explains why NK cell does not kill normal cells, such as erythrocytes, that do not express MHC-I on their surface but retain cytotoxic activity against MHC-I sufficient tumors (255). Elicitation of NK cell function is determined by the relative strength of activating and inhibitory receptor signaling and this concept, known as “*altered balance*,” ultimately controls NK cell activity under normal and disease conditions (256).

Decades of research in rodents have demonstrated the importance of NK cells in tumor clearance (14, 117, 247, 248). In humans, an 11-year follow-up study showed that low NK cell cytotoxic activity was correlated to an increased risk of cancer (257) and the presence of tumor-infiltrating NK cells is a positive prognostic marker for multiple malignancies including colorectal carcinoma (258), gastric carcinoma (259), and squamous cell lung cancer (260). Results from multiple studies demonstrate that NK cells have promise as a cancer immunotherapeutic for the treatment of hematological malignancies including acute myeloid leukemia and acute lymphoblastic leukemia (261–263). Allogenic NK cell therapy has proven effective in the clinic and, unlike T cell-based interventions, NK cell transfusion carries a relatively low risk of adverse off-tumor effects such as graft-versus-host disease (GvHD) (264).

Autologous NK cells may be inhibited by “*self*” MHC-I, thus limiting GvT effects in the absence of exogenous cytokines or antibodies (265, 266). Therefore, allogeneic NK cells along with hematopoietic stem cell transplant has been explored as a potential treatment for patients with high-risk solid tumors (263, 267, 268). Using non-myeloablative conditioning regimens to provide potent immune suppression without toxicity, the burden of cure then relies on the ability of transplanted donor cells to provide a GvT effect. Precedence in using low-intensity conditioning before transplanting allogeneic stem cells has been reported in Ewing sarcoma (269–271), osteosarcoma (272, 273), germ cell tumors (274), rhabdomyosarcoma (275–277), neuroblastoma (278–280), Wilms tumor (281), and CNS tumors (282), suggesting that alloreactive donor NK cells infiltrate heterogeneous solid tumors and cross the blood–brain barrier. A sizeable reduction in tumor burden has been observed (269). Using HLA-haploidentical family donors (parents and siblings), matched by only one HLA haplotype to the patient, have not only shown favorable outcomes in patients with solid tumors (263, 267, 283) but are also readily available and highly motivated donor sources. Thus, using HLA-haploidentical donors to augment GvT may be an effective strategy in patients undergoing allogeneic hematopoietic stem cell transplantation (HCT) for treatment of solid tumors (263, 284).

### Regulatory Functions of NK Cells

Most functions of NK cells are analogous to either CD8^+^ T or Th1 cells, including the production of pro-inflammatory cytokines (IFN-γ, TNF-α, and GM-CSF) and mediating cytotoxicity against infected or tumor cells (95). However, in addition to these, recent reports suggest NK cells also play regulatory functions (285, 286). NK cells mediate regulatory functions of other cell types including myeloid [DC (246, 287–290), monocytes (291–293), and macrophages (246, 294–296)] or lymphoid [T (297, 298) and B (299–301) cells] *via* cytokines production or through direct cell–cell contact in a receptor–ligand interaction-dependent manner. As part of the innate immune responses, effector functions of NK cells during the early phase is expected to dictate the threshold, direction, and the outcome of an immune response. These NK cell-mediated regulatory functions are predicted to occur during viral, bacterial, or protozoan infections, anti-tumor immune responses, unexpected immuno-pathological outcomes such as GvHD, and autoimmune diseases (302). Few of the examples are described below. A unique innate immunoregulatory function for the smaller CD56^bright^ subset of human NK cells (CD56^bright^CD16^dim^NKG2A^+^KIR^−^) was proposed due to their inherent ability to produce significant amounts of IL-10, and IL-13 along with IFN-γ, TNF-α, and GM-CSF compared to that of the more substantial CD56^dim^CD16^+^ subset (58). An Il-27-stimulated CD56^bright^CD16^dim^NKG2A^+^KIR^−^ subset was able to suppress the proliferation of autologous CD4^+^ T cells in patients with multiple sclerosis through a cytotoxic mechanism involving perforin (303) or by the release of Granzymes (304, 305). Importantly, CD56^bright^CD16^dim^NKG2A^+^KIR^−^ subset through their ability to produce adenosine and by the restricted expression of the ecto-nucleotide pyrophosphatase/phosphodiesterase 1 (CD203a/PC-1) and the nucleotide-metabolizing ectoenzyme CD38 (an NAD^+^ nucleosidase) was able to inhibit the proliferation of autologous CD4^+^ T cells (306).

Regulatory role of NK cells during GvHD is highly controversial (307). GvHD is one of the major complications and limiting factor in allogeneic HCT (308). Studies in both mouse and human lead to either suppressing or promoting rejection of HCT by NK cells. Furthermore, persistence or expansion of NK cells following HCT resulted in rejection and severe GvHD (309) while allograft-derived donor NK cells helped the engraftment of HCT by suppressing GvHD (310–313). Mechanistically, NK cells can help to contain GvHD through distinct mechanisms including the killing of professional APCs and thereby controlling the proliferation and expansion of graft-specific T cell (314, 315). In addition, NK cells were able to directly lyse graft-specific T cells following the expression of activating ligands of NKG2D on these T cell (316, 317). Expression of both mouse (316, 318) (H60a, H60b, H60c, Rae-1, and Mult-1) and human (319–321) (MIC-A, MIC-B, and ULBPs) activating ligands of NKG2D on stimulated T cells has been reported in a number of models. Also, shedding of these murine and human activating ligands has been demonstrated to employ a critical negative regulatory function on both T (322–324) and NK (325, 326) cells. These findings provide an exciting new avenue in understanding an inherent regulatory interaction between NK cell and APCs or T cells and thereby potential clinical utilization. Irrespective of the recent advances, the precise functions and associated mechanisms by which NK cells contribute to an immune-suppressive or immune-sufficient tumor microenvironment is far from fully defined. Similarly, the complex interplay of cytokines and ILs that are derived from and regulating the functions of NK and professional APCs during viral or bacterial infections is yet to be fully appreciated. Furthermore, defining the interactions between conventional NK cells (ILC1) and ILC2 or ILC3 can help to formulate better immunotherapeutic approaches to infections associated with mucosal tissues.

### NK Cells and CAR Therapy

Recent efforts to improve the clinical efficacy of NK cell immunotherapy has led to the development of genetically engineered NK cells that express a chimeric antigen receptor (CAR). Primary NK cells and NK cell lines can be engineered to express CARs which redirect the anti-tumor specificity of NK cells on an antigen-dependent basis (327). Through the manipulation of signaling motifs critical for lymphocyte activation, CARs are also designed to utilize specific intracellular signaling molecules which can further refine NK cell function and optimize their therapeutic potential (328, 329). Interestingly, the use of a clonal cell line derived from a human NK cell leukemia, known as NK-92, has been genetically modified to express fully functional CARs and these cells have shown great promise with regards to their safety and efficacy in recent clinical trials (327, 330, 331). Moreover, the use of irradiated cell lines may provide a fast and affordable off-the-shelf option for a personalized cellular immunotherapy treatment (332, 333) and are quickly rising to the forefront of cell-based cancer immunotherapies (Figure 9).

## Summary and Future Outlook

Natural killer cells possess promising potentials as a therapeutic tool to treat a number of maladies including malignancies (334). However, irrespective of their comparable ability in mediating anti-tumor cytotoxicity to that of CD8^+^ T cells, the clinical utilization of NK cells remains far from practical. In-depth understanding of NK cells at the single-cell transcriptomic landscape, methods to expand them *in vitro* without phenotypic and functional skewing, and detailed analyses of their *in vivo* longevity are central to facilitate the clinical utilization. NK cells regulate their effector functions utilizing both activating and inhibitory receptors (335, 336). Irrespective of our decades-long understanding, the precise intracellular signaling mechanisms by which NK cells discriminate the “*self*” from “*missing-self*” or “*non-self*” are still elusive. Emerging evidence suggests that mNK cells possess the ability to produce both pro-inflammatory to anti-inflammatory cytokines (159). However, the temporal regulation of these discrete functions is not yet fully understood. NK cells can be primed in response to a wide panel of ILs and other immunomodulatory factors (132, 158, 337). Our knowledge related to transcriptomic definitions of priming for an individual or combination of these priming factors is limited. NK cell subsets are comprised of a highly heterogeneous population (338). A pioneering study utilizing a novel technique known as mass-cytometry (CyTOF) determined that there are between 6,000 and 30,000 distinct NK cell phenotypes within a given individual based on unique combinations of 35 cell surface antigens (339). Studies on the genome-wide chromatin accessibility for regulomes provided similarities in regulatory circuitries of transcriptional programs between ILCs (ILC1 includes conventional NK cells) and CD4^+^ T helper subsets (340). However, the functional plasticity of subsets of NK cells yet to be fully appreciated. Controversies related to “adaptive” and “memory” characteristics of NK cells should be resolved by defining transcriptomic, genetic, and epigenetic alterations between naïve and “*antigen-experienced*” NK cells. Collectively, the future holds promising challenges to decipher new knowledge which will facilitate the utilization of NK cells for better therapeutic outcomes.

## Author Contributions

AA conceived and wrote the manuscript. CY contributed to the writing. MT edited the text. SM conceived, wrote, and edited the text and generated all the figures for the manuscript.

## Conflict of Interest Statement

The authors declare that the research was conducted in the absence of any commercial or financial relationships that could be construed as a potential conflict of interest.
